# Nutritional, Functional, Textural and Sensory Evaluation of *Spirulina* Enriched Green Pasta: A Potential Dietary and Health Supplement

**DOI:** 10.3390/foods11070979

**Published:** 2022-03-28

**Authors:** Deepak Kumar Koli, Shalini Gaur Rudra, Arpan Bhowmik, Sunil Pabbi

**Affiliations:** 1 Centre for Conservation and Utilisation of Blue Green Algae (CCUBGA), Division of Microbiology, ICAR—Indian Agricultural Research Institute, New Delhi 110012, India; mr.deepakkoli.iari@gmail.com; 2 Division of Food Science and Postharvest Technology, ICAR—Indian Agricultural Research Institute, New Delhi 110012, India; gaurshalini@gmail.com or; 3 Division of Design of Experiments, ICAR—Indian Agricultural Statistics Research Institute, New Delhi 110012, India; arpan.stat@gmail.com

**Keywords:** *Spirulina* enrichment, green pasta, sensory, flavonoids, antioxidants, phenolics

## Abstract

In house cultivated *Spirulina* powder was incorporated at 2 to 15% concentrations to enrich pasta prepared from semolina. *Spirulina* incorporation led to development of green color pasta with nutritional and functional fortification resulting in increase in its protein, total phenols, flavonoids, iron and calcium content by up to 77.47%, 76.62%, 162.88%, 296.99% and 57.27%, respectively, without causing detrimental changes to the textural and sensory attributes. FAME analysis revealed 2 to 2.5 times enhanced levels of γ-linolenic acid and docosahexaenoic acid in enriched pasta. Significant improvement in phenolics, flavonoids and antioxidant activity were also observed in comparison to control pasta. Analysis of theoretical and realized composition confirmed retention of nutrients post cooking revealing no significant loss in proteins and other nutrients. Principal components analysis demonstrated significant contribution of *Spirulina* to nutritional and functional attributes especially at higher concentrations. Pasta enriched with 12.5% *Spirulina* was rated as “liked very much” and the purchase intention was also high. *Spirulina* enrichment at concentrations above 10% (12.5%) with appreciable increase in nutritional and functional attributes without affecting textural or cooking quality and acceptable sensory evaluation can be a preferred alternative to augment health and prevent sickness. Since green color symbolizes freshness, hope, renewal and physical health, the consumption of *Spirulina* incorporated green pasta may be a potential option to enhance the livelihood and nutritional security of rural poor and a good alternative for hidden hunger alleviation programs for mass nutrition especially for infants and children in an effective manner.

## 1. Introduction

Microalgae have long been known to be well endowed to enhance the nutritional quality of conventional foods and positively affect human health owing to their high macro- and micro-nutrients content. Indeed, for thousands of years, edible microalgae *Arthrospira*, *Nostoc* and *Aphanizomenon* spices have been used for food [1]. Even though the first commercialized microalgae *Chlorella* and *Spirulina* as “health food” in Japan, Taiwan and Mexico emerged over 70 years ago [2], they still have not gained much ground in health foods segment owing to a peculiar flavor and aroma associated with them. Being a rich source of promising compounds with biological activity that could be used as functional ingredients, WHO has labeled them as super food. Owing to increasing awareness of health promoting nutrients and nutraceuticals necessary to ward off the lifestyle disorders, microalgae are receiving renewed attention in nutritional and food sciences. *Spirulina platensis* belonging to the family *Oscillatoriaceae*, is a multicellular and filamentous blue-green microalga (cyanobacteria) that thrives in warm, alkaline fresh-water bodies. The word “*Spirulina*” is derived from the Latin word meaning “helix” or “spiral”; denoting the physical configuration of the organism when it forms swirling, microscopic strands. This cyanobacterium contains 55–70% protein, 15–25% polysaccharides, 5–6% total lipids, 6–13% nucleic acids and 2.2–4.8% minerals [3]. Among the micronutrients, it contains a complex of vitamins A, B, D, E and K [4]. *Spirulina* is rich in minerals such as calcium, potassium, iron, nickel, chromium, magnesium, manganese, copper, sodium, zinc, selenium and lead along with carotenoids and essential fatty acids (3,6 γ-linolenic acid, α-linolenic acid, stearidonic acid, eicosapentaenoic acid, docosahexaenoic acid and arachidonic acid) necessary for a holistic balanced diet [5]. Besides, *Spirulina* is the richest natural source of digestible protein that offers all essential amino acids to the human body [6]. The higher digestibility of these microalgae is realized because of lack of cellulose in the cell wall of *Spirulina*, its wall material being composed of 86% digestible polysaccharides [7]. Contrary to misconception associated with them regarding the content of nucleic acids, in both *S. maxima* and *S. platensis,* the total nucleic acid content has been reported in the range of 4.2–6% on dry matter basis [8] which is well within safety norms, and thus do not pose any risk of rise in plasma uric acid upon consumption up to 10 g/day. Potential health benefits of *Spirulina* consumption include immunomodulation, antioxidant, anticancer, antiviral and antibacterial activities, as well as positive effects against malnutrition, hyperlipidemia, diabetes, obesity, inflammatory allergic reactions, heavy metal/chemical-induced toxicity, radiation damage and anemia without any toxicological effects [3]. Owing to these features, *Spirulina* is widely approved in several countries and has earned GRAS (Generally Recognized as Safe) status. It has been authorized by the FDA (Silver Spring, MD, USA) and The Brazilian Health Regulatory Agency (ANVISA, Brasília, Brazil) for consumption as food or food supplement. Since *Spirulina* cultivation requires high pH, it prevents the growth of other organisms [9], and that is why *Spirulina* has long been used as a dietary supplement by people living close to the alkaline lakes where it is naturally found. In many countries of Africa, it is still used as human food as a major source of protein and is collected from natural water, dried and eaten. Today, *Spirulina* is being used for self-health care strategy in many countries.

Most of the *Spirulina* available as dietary supplements is sold in the form of dried powder, capsules and tablets. Present forms of food aid by various agencies such as Algama, Phycon and few others, focus on fighting hunger as well as treating malnutrition especially in context of the needs of young children who are at maximum risk. There is an increasing demand for sophisticated products from microalgae as more consumers prefer to augment their health and prevent sickness, seek and try to incorporate these microalgae into their daily diet in the form of foods and/or ingredients [10] rather than the capsules or tablets. Attractive, more innovative products based on microalgae and/or their derivatives are positioning firmly in the food market.

Keeping in view, the high content of nutraceuticals present in *Spirulina* biomass, the new trend of research is inclined towards development of bioactive fortified functional food items consumed popularly such as doughnuts, pasta, salad dressing, mayonnaises and gelled-desserts. Batista et al. [11] have reported the enhancement of protein and functional properties of cookies along with increase in the total phenolic content and antioxidant capacity upon enrichment with different microalgae viz. *Spirulina platensis*, *Chlorella vulgaris*, *Tetraselmis suecica* and *Phaeodactylum tricornutum* at 2% and 6% levels. Santos et al. [12] developed chocolate flavor shake-type powdered food with *Spirulina* enrichment (750 mg/100 g of microalgal biomass) and found to contain 43.40, 41.34% protein, 45.73, 46.51% carbohydrates with 7.68 and 7.77 average acceptance rate for shakes with and without *Spirulina,* respectively. Recently, Lafarga et al. [13] formulated a broccoli soup enriched with different types of microalgae *Spirulina* sp., *Chlorella* sp., or *Tetraselmis* sp. at concentrations ranging from 0.5 to 2.0% (*w/v*) with incorporation effecting increase in phenolic, antioxidant activity and good consumer acceptance at correct formulation proportion. Addition of *Spirulina* at 2.6% concentration in snacks resulted in increased protein (22.6%), minerals (46.4%) and lipids (28.1%) without significantly affecting their sensory and physiological characteristics [14]. Rodríguez et al. [15] incorporated *Spirulina* biomass in fresh pasta, resulting in enhanced nutritional compositions, without affecting the sensory, textural and cooking quality. That *Spirulina platensis* could be used as an ingredient in the preparation of commercial unconventional dry pasta with acceptable sensorial and technological characteristics have been advocated by Pagnussatt et al. [16] too. Since pasta products are popular as convenience foods in tune with the requirements of modern life, their acceptance among consumers is mainly due to good sensory attributes, low cost, ease of preparation, amenability to suit various cuisines and longer shelf life. Pasta can also be employed in the preparation of different varieties of meals. According to the latest report by IMARC Group, the pasta market in India reached a sales value of US$335.4 million in 2018, exhibiting a compound annual growth rate (CAGR) of 17.1% during 2011–2018. Although many studies have investigated use of *Spirulina* in pasta, they pertain to levels between 2 to 10%. To the best of knowledge of authors, although hypothesized, no systematic studies exist on use of levels beyond 10% in pasta which were found acceptable in taste. Scientific records on textural and cooking profile of *Spirulina* enriched pasta are scanty and the detailed nutrient profile of cooked pasta is rarely reported. In this study we report the nutritive value of cooked pasta and losses upon cooking whatsoever. In view of the above observations, the aims of this research work were to: (i) evaluate the effect of *Spirulina* incorporation on the nutritive value and cooking attributes of green pasta; (ii) determine the consumer preferences for *Spirulina* supplemented pasta.

## 2. Material and Methods

### 2.1. Cultivation and Production of Spirulina

The *Spirulina* sp. CCC 540 was obtained from the germplasm collection of Centre for Conservation and Utilisation of Blue Green Algae (CCUBGA, New Delhi, India), Indian Agricultural Research Institute, New Delhi. India. The culture was maintained in Zarrouk’s medium [17] and its growth and purity were examined on regular basis. For experimental purpose, the *Spirulina* strain was cultivated in 100 mL autoclaved medium in 250 mL Erlenmeyer flasks in growth room at 28 ± 2 °C and light intensity of 55–60 μmol photon m^−2^ s^−1^ with photoperiod of 16 h:8 h light and dark rhythm. For large scale production of *Spirulina* biomass, it was cultivated in raceway ponds lodged in a semi-controlled Greenhouse using economical medium containing commercial fertilizers (Na_2_CO_3_ 9 g/L; Sufala, N:P:K (19:19:19) 1 g/L; NaCl 1 g/L; MgSO_4_ 0.2 g/L and single super phosphate 0.1 g/L). The cultures were circulated continuously by the paddle wheels at a flow rate of 20 cm/s. Culture depth was maintained at 10 cm in the ponds. The culture was harvested after 21–25 days of growth, filtered, washed properly, sun dried and stored at room temperature (25 ± 2 °C) until further use within a week.

### 2.2. Pasta Preparation

**Figure 1:**
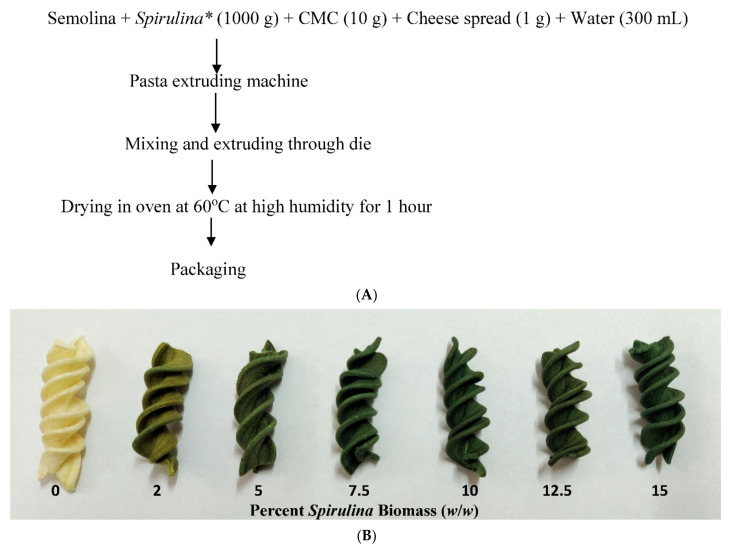
(**A**). Flow chart for preparation of *Spirulina* enriched pasta. * *Spirulina* addition shall vary with its percentage enrichment in total mixture. (**B**). Fusilli pasta (control and enriched green pasta with increasing order of *Spirulina* biomass) (*w/w*). Supplementation with edible blue green alga, *Spirulina* provided an appealing green tone that varied with *Spirulina* concentration.

Pasta elaboration into fusilli shape (Figure 1A) was performed using laboratory scale cold extrusion equipment (Dolly, La Montiferrina, Cittadella, Italy). Fresh pasta was prepared using commercial *durum* wheat semolina, water, carboxymethyl cellulose (1%, fl. basis), cheese spread (0.1%, Mother Dairy, Delhi, India) *Spirulina* biomass 2, 5, 7.5%, 10%, 12.5% and 15% (*w/w*, *n* = 3) and dehydrated in hot air oven at 60 ± 2 °C under high humidity until attainment of 7–8% moisture (Figure 1B). For analysis, 250 g samples of each group were packaged in LDPE packets and stored in a dark place at room temperature (25 ± 2 °C) until analysis.

### 2.3. Nutritional Evaluation

Proximate analysis (fat, fiber, ash and protein) of both raw and cooked pasta was performed using AOAC (Association of Official Analytical Chemists, Rockville, MD, USA) methods. The protein content of pasta was calculated by determining nitrogen using micro Kjeldahl method [18] with fully automatic digester and distillation unit (Velp Scientifica, Usmate, Italy) and multiplying by factor of 6.25 [19]. The crude lipids content was estimated by using chloroform: methanol extraction method [20]. Derivatization of total lipids as fatty acid methyl esters (FAME) was performed according to Ichihara and Fukubayashi [21] the fatty acids profile for each of the pasta samples was determined by Gas Chromatography (Shimadzu GC-2014, Serial no.—C121652, Kyoto, Japan) on Restek Stabilwax column (30 m, 0.25 mm ID) using flame ionization detector (FID, Shimadzu, Kyoto, Japan). The carbohydrates content was determined by Anthrone method [22]. Calorific value of pasta was calculated based on the composition, using the Atwater conversion factors of 4 kcal/100 g for protein and carbohydrates, and 9 kcal/100 g for lipids [23]. Moisture content was determined by weight difference method upon drying in the oven at 100 °C [18] and ash content was determined using muffle furnace [18]. Total phenolic content of pasta samples was estimated spectrophotometrically (Varian Cary, Melbourne, Australia) using Folin–Ciocalteu reagent [24], and results were expressed as Gallic Acid Equivalent (mg GAE 100 g^−1^). Flavonoids content estimation was performed with spectrophotometric determination using aluminum chloride solution [25] and results were expressed as pmol Quercetin Equivalent per gram (QE/g). Antioxidant activity was determined using two in vitro assays, namely: Ferric Reducing Antioxidant Activity (FRAP) according to Benzie and Strain [26]; and 2, 2-diphenyl-1-picryl hydrazyl (DPPH) assay as specified by Apak, Güclü et al. [27]. The results were expressed as μmol Trolox Equivalents (TE) 100 g^−1^. All reagents and chemicals used for the study were of analytical grade from SRL chemicals, Mumbai, India.

### 2.4. Minerals

Pasta samples were digested with di-acid (HNO_3_: HClO_4_ 4:1). The wet digest was made up to 100 mL with double distilled water and filtered with Whatman 42 paper (pore size 2.5 μm). Iron and zinc content were estimated through atomic absorption spectrophotometer (Z-Xpress 8000, Spectrum Instruments GmbH, Überlingen, Germany ). Calcium content in the digested pasta sample was determined using flame photometer [28].

### 2.5. Cooking Quality of Pasta

Cooking quality of pasta was determined following protocols of AACC method 16–50 [29]. Optimum cooking time (min) for each sample was determined by observing disappearance of white core after the pasta was pressed between two Plexiglass plates. Cooked weight (g), as a measure of the degree of pasta hydration taking 10 g dry pasta sample was recorded. Cooking loss (weight of total solids lost in cooking water, expressed as percent), was measured by evaporating the pasta cooking water to dryness in an oven set at 100 °C.

### 2.6. Textural Analysis

The textural attributes of cooked pasta viz., firmness, stickiness upon compression and cutting force were measured on Texture Analyzer (TA-XT2; Stable Micro Systems Ltd., Surrey, UK) using software Exponent (6.1). Texture of the cooked pasta samples was measured using Texture Analyzer using 5 kg load cell, and settings as: pre-test speed 2.0 mm/s, test speed 0.5 mm s^−1^, post-test speed 0.5 mm s^−1^, strain 90% in compression mode (return mode) with 36 mm radiused cylindrical probe. The maximum force in force-time graph was taken as firmness and the negative force was noted as stickiness. Five measurements were taken for each sample. Cutting force for cooked pasta was determined by HDP/BS probe of TAXT2 Texture Analyzer for three cooked pasta singlets kept side by side under the blade with test speed 3 mm/s and distance 20 mm in compression mode. The peak positive force determined using texture exponent software was noted as cutting force (g).

### 2.7. Sensory Evaluation

Eighty-three semi-trained panelists (58 males and 25 females), aged between 21 to 45 years, ranked the freshly cooked pasta served in random order two hours post meal. All panelists were briefed about the product and health benefits of *Spirulina*. All of them were regular consumers of pasta and knew about desirable quality attributes. Sensory evaluation of cooked pasta was performed based on 9-point hedonic scale (0 = not acceptable; 9 = highly acceptable). Intention to purchase the pasta was also asked from the panelists in the proforma. Sensory evaluations were conducted in the isolated booths at room temperature (25 ± 2 °C). Between two samplings, the panelists were provided with plain water to rinse their mouth.

### 2.8. Statistical Analysis

All analysis was conducted in triplicates. Test of significance when the *p*-value was <0.05, was calculated with Tukey’s HSD test for comparison of treatment effects for each of the parameters by SPSS-16.0 statistical package. Principal Components Analysis (PCA) was carried out to determine multivariate relation among varying concentrations of *Spirulina* supplementation on nutritional parameters, functional attributes and antioxidant activity of pasta. It was also analyzed through R version 3.6.2 (12 December 2019)—Copyright © 2019 The R Foundation for Statistical Computing (Vienna, Austria), Platform: x86_64-w64-mingw32/x64 (64-bit). Dendrogram (UPGMA) was also prepared to confirm the grouping of the different parameters.

## 3. Results and Discussion

### 3.1. Proximate Composition of Spirulina Platensis

*Spirulina* sp. CCC 540 was found to contain 65.71% protein, 6.94% total lipids, 21.87% carbohydrates, 8.34% ash and 26.59, 107.83 and 1.87 mg/100 g of iron, calcium and zinc, respectively (Table 1). Our in-house *Spirulina’s* composition was in range with *Spirulina* powder used by Tańska et al. [30] for preparation of corn extrudates which contained 60.7% protein, 7.2% fat, 15.2% carbohydrates and 7.9% ash. *Spirulina platensis* cultivated in our lab was found superior over the culture used by El-Hameed et al. [31] for spaghetti preparation; it also contained similar range of nutrient composition (53.92% crude protein, 6.67% total lipids, 18.10% carbohydrates and 13.11% ash).

### 3.2. Nutritional Composition of Spirulina Enriched Pasta (SEP)

Significant variation was observed for protein, total lipids and micronutrients content of different pasta formulations (Table 2).

#### 3.2.1. Proteins

**Figure 2:**
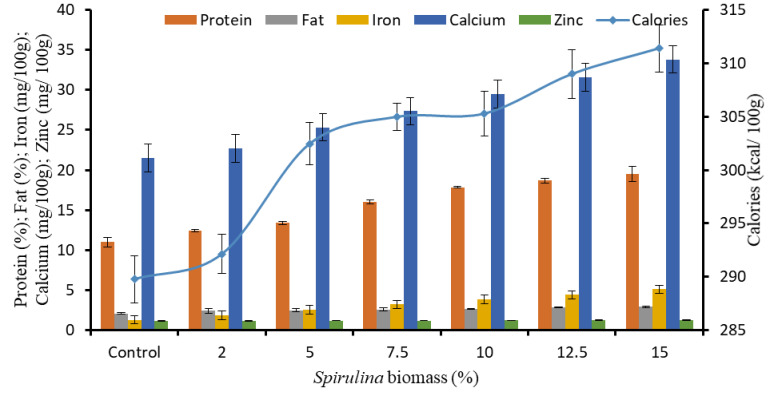
Proximate and mineral composition of cooked pasta enriched with different concentration of *Spirulina* biomass.

Protein content of semolina pasta was 11.52% and upon enrichment with *Spirulina* at various levels (2%, 5%, 7.5%, 10%, 12.5% and 15% w/w), increase in protein content by up to 77.47% was recorded. The augmentation in protein content with *Spirulina* addition is in conformity with studies conducted by several researchers [32,33]. The theoretical protein content in SEP12.5 (most acceptable formulation) was envisaged to be 19.74% but the realized value in cooked samples was 18.66% confirming that most of the nutritional qualities of *Spirulina* remained intact in final product. Cooked pasta samples recorded a marginal decrease in the protein content due to leaching in the gruel and lay in the range of 11.00–19.51% on dry weight basis (Figure 2) as against 11.52–20.44% in uncooked pasta (Table 2). Considering serving size of dry pasta as 85 g, a single serve of SEP12.5 upon cooking provides 15.86 g of protein which is 26.4% of recommended protein intake (60 g per day for adults, Dietary Guidelines for Indians, National Institute of Nutrition, India) [34]. Hence this pasta can qualify for high protein foods category (section 101.54 of FDA food labeling norms, [35]).

#### 3.2.2. Fats

Increase in lipid content was also reported for SEP over Semolina pasta, containing 2.99% crude fat. The increase in lipids content ranged from 4.53 to 37.60% (Table 2). Lucas et al. [14] developed snacks with addition of *Spirulina* biomass at 2.6% concentration and reported an increase in lipid, protein and minerals content by 28.1%, 22.6% and 46.4%, respectively, over control snacks. Tańska et al. [30] also reported an increase in protein (4%), total lipids (0.4%), ash (0.4%) and fiber (0.7%) with 8% *Spirulina* addition in corn extrudates. While raw pasta enriched with 12.5% *Spirulina* had 3.94% lipids, its cooked form had 2.8% lipids on dry weight basis (Figure 2). Our findings are in agreement with another report by Agustini et al. [36] that there was a decrease in total lipids content of the noodles post cooking. Further, the fatty acid analysis of the pasta samples revealed variation in amongst samples with respect to proportion of *Spirulina* biomass used for enrichment. Major fatty acids reported from *Spirulina* sp. CCC 540 in increasing number of carbons were: myristic acid (MA, C14:0): 0.40%, myristoleic acid (MTA, C14:1): 0.47%, palmitic acid (PA, C16:0): 41.10%, palmitoleic acid (PTA, C16:1): 4.41%, stearic acid (SA, C18:0): 1.28%, oleic acid (OA, C18:1): 25.48%, linoleic acid (LA, C18:2): 5.88%, arachidic acid (ARA, C20:0): 0.52%, γ-linolenic acid (GLA, C18:3): 9.25% and docosahexaenoic acid (DHA, C22:6): 0.59% of total fatty acids. As per Almahrouqi et al. [37] fatty acid profile of *S. platensis* at different salinity were reported as: PA (54.78–57.25%), PTA (2.47–2.74%), Heptadecanoic acid (0.14–0.23%), SA (0.96–1.27%), OA (3.19–3.77%), LA (23.84–24.94%), GLA (11.55–12.81%) and Eicosadienoic acid (0.22–0.31%). The applications of gamma-linolenic acid (GLA) and docosahexaenoic acid (DHA) and other poly unsaturated fatty acids in the treatment and prevention of gastrointestinal cancer and cardiovascular disease have been widely reported [38]. Thus, their enhanced content in *Spirulina* enriched pasta (SEP) points towards the nutraceutical potential of this preparation. A sequential increase was reported in MTA, PA, PTA, LA, ARA, GLA and DHA, while MA, SA and OA were found to decrease. Raw SEP12.5 was found to contain 0.81%, 33.94%, 3.98%, 12.25%, 1.97%, 0.33 and 0.59% MTA, PA, PTA, LA, ARA, GLA and DHA of total fatty acids, respectively. GLA and DHA were reported to increase up to 252.64% and 222.98% of total fatty acids in SEP12.5 in comparison to control.

Thus, a single serve of cooked SEP12.5 pasta can provide 19.75 mg DHA and 11.05 mg GLA. Considering recommended EPA+DHA and GLA amounts of 500 mg and 1.4 g/day [39,40], this amounts to 10% of recommended intake. Vatsala and Sudesh [41] have reported the value of γ-linolenic, palmitic and stearic acid in the range of 0.70–2.10%, 40.48–42.00% and 17.00–18.40% of total fatty acids upon enrichment of noodles with 2%, 4% and 6% *Spirulina* biomass. El-Hameed et al. [31] have also reported that the increase in concentration of *Spirulina platensis* from 0.5–2% led to increase of fatty acids (palmitic, margaric, stearic, oleic and γ-linolenic acid) in spaghetti. To the best of the knowledge of authors, this is the first report of GLA and DHA content in raw and cooked *Spirulina* enriched pasta products.

#### 3.2.3. Carbohydrates

The carbohydrates content in control pasta was estimated to be 71.68% (Table 2). Upon enriching pasta with *Spirulina* biomass (21.87% carbohydrates) at different levels, a proportional decrease in carbohydrates’ content was reported. SEP12.5 and SEP15 were found to contain 65.84% and 63.49% carbohydrates, respectively. Our findings are in consonance to Fradique et al. [42] who have also reported almost 10–12% decrease in carbohydrates upon incorporation of *Spirulina* in pasta at 2% level. Similarly, Tańska et al. [30] reported a decrease in carbohydrates content from 79.4 to 74.3% in control and *Spirulina* enriched corn extrudates, respectively. Reduction in carbohydrates content upon cooking ranged from 18.31 to 21.51%. Carbohydrates content for cooked control, SEP12.5 and SEP15 was found to be 56.81%, 52.29% and 51.87%, respectively.

#### 3.2.4. Micronutrients

The cultivated *Spirulina platensis* had 26.59, 107.83 and 1.87 mg/100 g of iron, calcium and zinc, respectively, on dry weight basis. While control pasta had 1.28, 21.57 and 1.13 mg/100 g of iron, calcium and zinc, their content in SEP increased up to 296.99%, 57.27% and 10%, respectively (Table 2). These findings are similar to those of Burcu et al. [43] who supplemented bread with 10% *S. platensis* and reported an increase of 45.95, 14.0 and 3.24 mg/100 g of calcium, magnesium and iron content, respectively, over control. Several researchers [16,44] have also reported similar findings for *Spirulina* enriched pasta, biscuits or cookies. No significant differences were recorded between minerals content of cooked and raw pasta, indicating non-significant leaching of minerals in cooking water (Figure 2). A single serve of cooked SEP12.5 pasta could provide 19.68% of RDA for iron and 11.66% of zinc. Its label can therefore claim “good source of iron” on the package. Further, it provides calcium content of 26.86 mg per serving. SEP can therefore emerge as a suitable means for hidden hunger alleviation programs.

On the basis of the nutrients’ composition, the calorific value per 100 g of pasta was calculated in the range of 359.71 to 372.75 calories which was 4.12% more in SEP12.5 pasta when compared with control (Table 2). These findings are on anticipated lines as *Spirulina* is a rich source of protein with low lipids and low calories content and is cholesterol-free unlike the other protein sources viz. dairy and meat products [45]. The ash content in control pasta was estimated to be 0.57% while that of SEP ranged from 1.04 to 1.39%. SEP12.5 and SEP15 were found to have 1.34% and 1.39% ash content (Table 2), respectively. Agustini et al. [36] have reported profound increase in the ash content for *S. platensis* enriched dried noodles (2.46%) as compared to control noodles (1.37% ash). No significant difference was recorded in the initial moisture content (5.52–6.21%) of the control and SEP (Table 2).

#### 3.2.5. Principal Component Analysis

**Figure 3:**
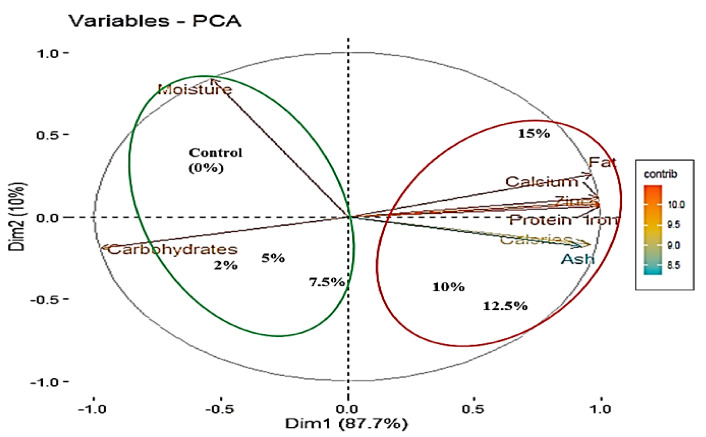
Results of PCA of data on the effect of supplementation of pasta with different concentrations of *Spirulina* biomass (%) on nutritional parameters presented as scatter biplots.

**Figure 4:**
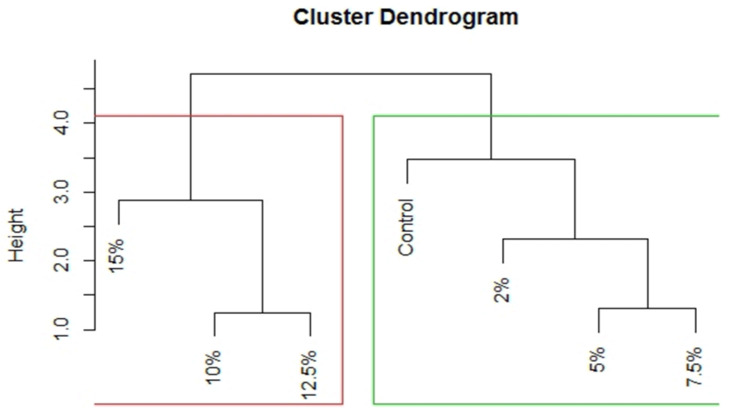
Unweighted pair group method with arithmetic mean (UPGMA) depicting the grouping based on all the nutritional composition parameters.

Principal Component Analysis (PCA) was carried out to establish correlation among different nutritional parameters (Table 3) due to change in *Spirulina* biomass supplementation of pasta. Out of the six Dimensions (Dim) or Principal Components (PC), first PC alone accounted for 87.65% variation and second PC for 10% variation. These two PCs together explained 97.72% variation in the data. Thus, these two PCs were screened out for further interpretation. All the variables except moisture were having high loading on Dim 1 whereas moisture was having high loading on dimension 2. Besides, carbohydrates and moisture were having negative impact on dimension 1. The PCA results also produced contribution values of variables under study. Based on Figure 3 it could be interpreted that treatments are having significant impact on almost all the variables (which are lying above the cutoff line of expected contribution of variables) except calories and ash. From the biplot (Figure 3) obtained through PCA method based on contribution of variables towards Dim 1 and 2, it was clear that, two groups can be formed where Group 1 treatments viz. 15%, 12.5% and 10% supplementations were having measurable impacts on all the nutritional composition parameters which were significant except moisture and carbohydrates. On the other hand, Group 2 treatments viz. 7.5%, 5% and 2%, including control (0%), mainly impacted moisture and carbohydrates. Dendrogram (UPGMA) which is a simple agglomerative hierarchical clustering method based on Euclidian distance also revealed the same grouping (Figure 4).

### 3.3. Total Phenolics, Flavonoids and In Vitro Antioxidant Activity

**Figure 5:**
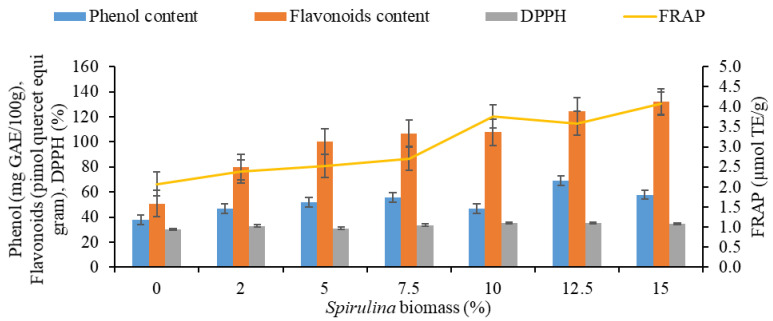
Total phenolic, flavonoid and antioxidant content of cooked pasta enriched with different concentration of *Spirulina* biomass.

Total phenolics, flavonoids which represent the functional component of a food material were found to be higher in SEP as compared to control pasta. The total phenolics content (TPC) of pasta ranged from 50.25 to 88.75 mg GAE 100 g^−1^ increased from 8.71 to 76.62% after incorporation of *Spirulina* biomass (Table 2). Phenolics as secondary metabolites are major health promoting compounds and contribute to antioxidant activity of microalga and subsequently, preparations supplemented with microalgae. These phenolic compounds are known to scavenge reactive oxygen species (ROS) and free radicals which are harmful to the human body. The raw and cooked SEP12.5 were found to have 81.75 and 69.05 mg GAE/100 g TPC. These values are comparable to that provided by strawberry juice (81 mg GAE/100 g) [46], considered as good source of polyphenolics. Flavonoids content of raw SEP12.5 was found to decrease from 144.25 to 124.55 picomol QE/g after cooking. The flavonoids provided by the SEP are also in good proportion and has potential for functional use. Flavonoids are considered to possess anti-inflammatory, anti-proliferative, hepato-protective, antiviral activities and help to protect from cancer, cardiovascular and age-related diseases. Increase in TPC from 0.72 mg GAE/g to 1.89 mg GAE/g upon enrichment with *Spirulina* powder at 6% level in bread has also been reported by Vatsala and Sudesh [47]. Kumar et al. [48] prepared *Spirulina* enriched bars whose TPC ranged from 5.56–7.90 mg GAE/g compared to 0.98 ± 0.03 mg GAE/g in control. Increment in TPC is in accordance with the previous reports [15,49] who have developed various *Spirulina* enriched products. Flavonoids content for SEP ranged from 92.06 to 167.31 pimol QE/g when compared to the control (63.65 pimol QE/g). SEP12.5 and SEP15 recorded 126.64% and 162.88% increase in flavonoids content, respectively (Table 2). After cooking of the pasta samples, the decrease in TPC and flavonoids content was recorded in the range of 37.93–57.78 mg GAE 100 g^−1^ and 50.69–132.10 pimolQE/g, respectively (Figure 5). Similar findings have been reported owing to degradation of a specific fraction of phenolics after heat treatment during cooking or leaching in boiling water [50].

**Figure 6:**
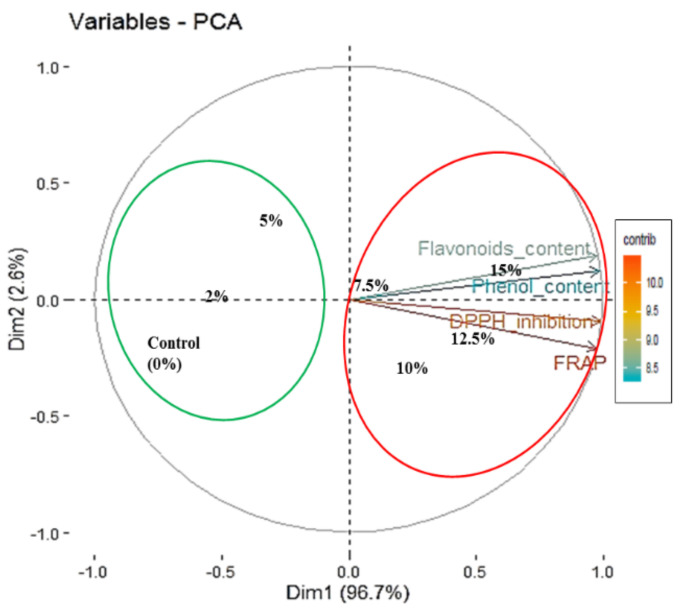
Results of PCA of data on the effect of supplementation of pasta with different concentrations of *Spirulina* biomass (%) on antioxidant and functional properties/parameters presented as scatter biplots.

**Figure 7:**
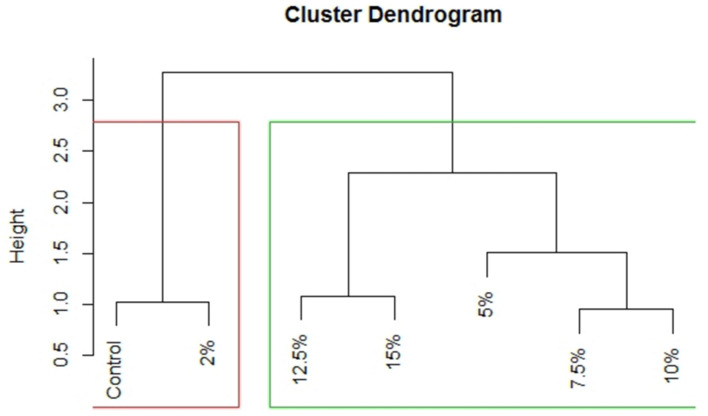
Unweighted pair group method with arithmetic mean (UPGMA) depicting the grouping based on functional and antioxidant parameters.

The percentage inhibition of DPPH and FRAP measured the free radical scavenging attributes of the SEP and indicate its antioxidant potential. The DPPH inhibition of pasta ranged from 42.80 to 50.69% and FRAP ranged from 2.52 to 5.12 μmol TE g^−1^. Significant improvement in both the parameters was observed in comparison to control pasta, and maximum DPPH inhibition and FRAP activity was recorded for SEP15 (50.69% and 5.12 μmol TEg^−1^) followed by SEP12.5 (49.37% and 4.62 μmol TE g^−1^: Table 2). DPPH free radical scavenging activity varies in proportion to the phenolics content in a particular substance [49]. The addition of *Spirulina* biomass significantly improved the antioxidant capacity in terms of DPPH scavenging activity with nearly four times increase even upon enrichment at 5% level from the control pasta [51]. Similar trend of increased DPPH activity has been reported in the range of 11.90 to 16.61% in bread prepared with varied concentrations (2–6%) of *Spirulina* biomass which also exhibited increased phenolic content [47]. Rodríguez et al. [15] prepared bread wheat pasta with 5, 10 and 20% of *Spirulina* addition along with control and reported FRAP activity viz. 1.93, 2.87, 4.24 and 1.16 μmol TE g^−1^, respectively. The DPPH scavenging activity and FRAP activity after cooking were recorded in the range 30.10–35.19% and 2.07–4.08 μmol TE g^−1^, respectively. After cooking, DPPH scavenging activity and FRAP activity was recorded for SEP15 (34.73% and 4.08 μmol TE g^−1^) and for SEP12.5 (35.16% and 3.59 μmol TE g^−1^: Figure 5). The maximum “%-scavenging of DPPH-radical” up to 50.69% was obtained at SEP15, which indicates the proton donating ability of *Spirulina*. The result of DPPH assay signified that the *Spirulina’s* antioxidant virtue also works well in hydrophobic environment whereas, the FRAP and reducing power assay signified the possible presence of a high amount of amino acids that have reducing ability. The positive results in DPPH together with FRAP and reducing power assays suggest that the *Spirulina’s* antioxidant activity is contributed by both through scavenging the already produced ROS via redox reaction and by reducing the oxidized-metal ions, which can promote more ROS production. In case of PCA of antioxidant and functional activity related parameters, first PC alone explains 96.7% variation in the data i.e., all the variables are having high loading on Dim 1 or PC1 (Table 4). Based on Figure 6, it can be inferred that treatments are having significant impact on DPPH inhibition and FRAP (which are lying above the cutoff line of expected contribution of variables). From the biplot (Figure 6) obtained through PCA method based on contribution of variables towards Dim 1, it is clear that, again two groups can be formed; Group 1 with 15%, 12.5%, 10%, 7.5% and 5% of treatments, respectively, having measurable impacts on antioxidant activity as measured by DPPH inhibition and FRAP whereas Group 2 consisting of only 2% and control (0%) treatments which will be having not that much impact on the characters under study. In this case also the dendrogram (UPGMA) prepared based on Euclidian distance reveals the same grouping (Figure 7).

### 3.4. Cooking Quality

Optimum Cooking Time (OCT) of pasta was 5.35 and 5.21 min for control and SEP12.5, respectively, (Table 5) showing marginal change. Fradique et al. [42] also reported no change in OCT and textural properties in fresh spaghetti prepared with addition of *Spirulina maxima* and *Chlorella vulgaris*. The lower OCT could be due to the fact that proteins present in *Spirulina* hindered the development of gluten network, therefore, the cooking time reduced with increase in *Spirulina* biomass after initial increase in OCT [52]. Increase in true volume, increase in weight and cooking loss are main attributes determining the physical changes occurring during cooking of pasta. These are important indicators which also determine the quality of pasta prepared. Increase in length and width of 23.76% and 58%, respectively, was recorded for SEP12.5 post cooking as compared to control (30.97% and 47.11%: Table 5). True volume of pasta as determined by volume displacement of toluene recorded increase in the range of 70.67 to 96.67% in SEP as compared to control (100%: Table 5). The true volume increase was found to be significantly lower for SEP as compared to control. SEP pasta was also found to have higher increment of weight (151.95 to 193.99%) after cooking over control (137.82%). Fradique et al. [42] have also reported higher weight of SEP upon cooking owing to high hydration of the starch present in the product in the presence of weaker protein network. Similar observations for pasta enriched with microalgae have been reported by Prabhasankar et al. [53]. The gruel loss of control and SEP was found to lie in the range of 4.13% (control pasta) to 6.66% (Table 5). The gruel loss was below the technologically acceptable limit (≤8%) [54] for all the pasta samples. In particular, SEP12.5 and SEP15 recorded cooking loss of 6.66% and 6.22%, respectively. The increase in cooking loss upon *Spirulina* enrichment can be related to steric hindrance in the gluten-protein network that is responsible for physical integrity of product during cooking [55]. The reinforced dough matrix of microalgae proteins and gluten is able to entrap starch and that may be a possible reason for cooking loss in a technologically acceptable limit. The recorded cooking loss for SEP was found to be lower than that reported by Özyurt et al. [44] which was in the range of 6.83 to 7.84%.

### 3.5. Textural Analysis

There were significant variations in textural attributes viz. firmness, stickiness, cut force, consistency and cohesiveness of cooked pasta with varied levels of *Spirulina* biomass supplementation. The mean firmness and cut force varied from 2 596.45 to 4912.19 g and 531.78 to 637.28 g, respectively, across different preparations of pasta (Table 6). Firmness of cooked pasta is one of the most important parameters governing its acceptability. It is analyzed by measuring the resistance offered by the pasta upon compression. The increasing proportion of *Spirulina* biomass in the matrix is certain to hamper the cross-linkages of gluten responsible for binding starch–protein complexes together. However, the decrease in firmness should not be so excessive that the structure of pasta becomes lumpy and unable to hold itself. In the present study, as anticipated, firmness of SEP was lower than control, however, no systematic trend was recorded with increase in biomass level in cooked pasta. Cutting force, another major indicator of acceptability of pasta is the force required by knife or fork to cut the cooked pasta and comes closer to the actual process of consuming the pasta as compared to firmness, which resembles the process of bite post-consumption [56]. Cut force of control pasta recorded as 637.28 g was found higher than SEP at all concentrations. However, an interesting trend reversal was observed beyond biomass level of 10% where increase in cut force was observed for SEP 12.5 and SEP 15. It is possible that the proteins and dietary fiber contributed by *Spirulina* reached the threshold level of interaction above 10% and were able to forge intra- and inter-constituent linkages promoting the cohesion and resulted in higher cut force.

Stickiness of pasta is another important attribute to measure the *al dente* feeling upon consumption of pasta. Stickiness of control pasta was found to be lower than SEP, however, again no definite trend was observed with increment in level of *Spirulina* biomass in line with other textural characters. The increment in stickiness was found to vary from 6.27 to, as high as, 78% (Table 6). This increase is again on expected lines as *Spirulina* possesses soluble polysaccharides or dietary fibers. Depending on the complex interaction during cooking process between fiber-starch and available water for hydration, the soluble fibers are able to hydrate and exert adhesive/stickiness attribute to the cooked pasta. The consistency of cooked pasta generally relates to the smooth texture and stickiness of pasta. While consistency of control pasta was recorded to be 23.98 kg.s., the SEP recorded lower consistency values of 13.53 kg.s. and 15.52 kg.s. for SEP12.5 and SEP15, respectively, indicating partial disruption of gluten network owing to interference by biomass constituents (Table 6). Though the consistency of SEP pasta was lower than control, no systematic trend was observed with increase in proportion of *Spirulina* biomass in semolina matrix. These findings hold relevance as the water holding capacity of the product also increases with increase in biomass that helps to soften the structure of the formulated product [57]. Thus, even though the network formation is not disrupted, beyond a threshold limit, which in this case was above 12.5%, the moisture uptake might be comparatively high for the structure to support and strengthen. Despite the anomaly, the firmness, cut force and other textural properties of pasta are perceived by consumers and pervasively have been found to have negative correlation with cell structure and expansion of the product [32].

### 3.6. Sensory Attributes

**Figure 8:**
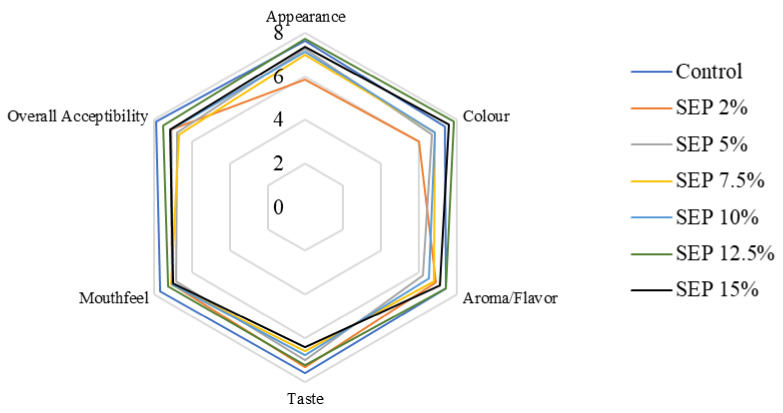
Acceptability scores of pasta containing different levels of *Spirulina*.

Figure 8 depicts the average findings on sensory attributes following evaluation by 83 panelists. The addition of *Spirulina* in pasta led to development of bright green color that improved its appearance. Similarly, other parameters were in the range of “Like moderately” for degree of acceptance. SEP12.5 was rated as “Like very much” among all other formulations. The average overall score of acceptability was reported highest for control pasta (7.91) followed by SEP12.5 (7.56). Further, the appearance, color and aroma of pasta enriched with *Spirulina* were found better than the control. In terms of color, appearance and flavor, also SEP12.5 was rated high (7.81, 7.82 and 7.48, respectively). Further, the purchase intention of the pasta was high (approximately 8.13) implying that the evaluators “probably would buy” the evaluated products if offered in the market. Our findings are contrary to Morsy et al. [32] who reported that sensory acceptance for snack food formulated with 12.5% *Spirulina* powder decreased. It could be made possible by the incorporation of cheese in the pasta which was able to mask the flavor of *Spirulina*. Muresan et al. [51] have also revealed that the nutritional value and sensory attributes of pasta was significantly improved by addition of *Spirulina* (2% and 5%). Ours is the first study to have overcome the flavor barrier for *Spirulina* at such high concentrations. Earlier, a study performed by Burcu et al. [42] revealed the taste, smell and aroma attributes of bread enriched with 10% *Spirulina* biomass as “consumable” during panelist’s evaluation.

## 4. Conclusions

Results reveal that green pasta prepared with supplementation of *Spirulina* biomass submitted significantly higher in chemical composition such as protein, minerals, ash and total lipids over the control pasta. Besides, pasta enriched with *Spirulina*, at different levels of addition, possessed higher phenolic, flavonoids and antioxidants activity over control. *Spirulina* biomass supplementation above 7.5% was more effective in enhancing the nutritional value of pasta and accounted for higher positive influence on antioxidant activity and functional properties expressed in terms of phenolics and flavonoids. The pasta cooking quality was not affected adversely by incorporation of microalgae in the composition of pasta, and the textural properties were also favored that represent acceptable technological characteristics. Although, all the pasta samples were favorably evaluated for sensory attributes, pasta enriched with 12.5% *Spirulina* biomass was rated most acceptable by the panelists. Therefore, green pasta enriched with *Spirulina* shall surely have a positive impact on the consumer and be considered as a feasible and attractive option for improving the nutritional health of the population besides providing the industry with a diversified functional product.

## Figures and Tables

**Table 1:** Proximal composition of biomass of *Spirulina* sp. CCC 540 (means ± SD).

Constituent	Amount
Moisture (%, db)	5.81 ± 0.17
Energy (kcal in 100 g)	412.78 ± 11.04
Carbohydrates (%, db)	21.87 ± 0.44
Protein (%, db)	65.71 ± 1.00
Fat (%, db)	6.94 ± 0.55
Ash (%, db)	8.34 ± 0.06
Iron (mg/100 g)	26.59 ± 0.80
Calcium (mg/100 g)	107.83 ± 2.50
Zinc (mg/100 g)	1.87 ± 0.03

All values are provided on dry weight basis (db) and are average values for analysis in triplicates.

**Table 2:** Nutritional, phenolic, flavonoids and antioxidant content of different formulations of *Spirulina* biomass enriched pasta.

Component	Control	2%	5%	7.50%	10%	12.50%	15%
Moisture (%)	6.21 ± 0.182 ^a^	5.89 ± 0.135 ^abc^	5.89 ± 0.215 ^abc^	5.72 ± 0.212 ^abc^	5.67 ± 0.016 ^bc^	5.52 ± 0.021 ^c^	6.05 ± 0.025 ^ab^
Energy (kcal in 100 g)	359.71 ± 2.74 ^c^	360.52 ± 1.81 ^c^	367.14 ± 1.45 ^b^	369.80 ± 1.61 ^b^	371.93 ± 2.37 ^b^	374.54 ± 1.19 ^b^	372.75 ± 2.47 ^b^
Carbohydrates (%)	71.68 ± 1.48 ^b^	70.30 ± 0.73 ^b^	69.64 ± 0.45 ^bc^	68.43 ± 0.83 ^bcd^	66.62 ± 0.18 ^cde^	65.84 ± 1.00 ^de^	63.49 ± 1.09 ^e^
Protein (%)	11.52 ± 0.38 ^g^	12.80 ± 0.13 ^f^	14.58 ± 0.10 ^e^	16.07 ± 0.13 ^d^	17.97 ± 0.31 ^c^	18.93 ± 0.39 ^b^	20.44 ± 0.18 ^a^
Fat (%)	2.99 ± 0.14 ^b^	3.12 ± 0.86 ^ab^	3.36 ± 0.26 ^ab^	3.53 ± 0.22 ^ab^	3.73 ± 0.25 ^ab^	3.94 ± 0.11 ^ab^	4.41 ± 0.10 ^a^
Ash (%)	0.57 ± 0.002 ^e^	1.04 ± 0.030 ^d^	1.13 ± 0.034 ^cd^	1.12 ± 0.066 ^cd^	1.23 ± 0.006 ^bc^	1.34 ± 0.005 ^ab^	1.39 ± 0.023 ^a^
Iron (mg/100 g)	1.28 ± 0.003 ^g^	1.81 ± 0.006 ^f^	2.55 ± 0.007 ^e^	3.19 ± 0.008 ^d^	3.86 ± 0.007 ^c^	4.44 ± 0.013 ^b^	5.081 ± 0.004 ^a^
Calcium (mg/100 g)	21.57 ± 0.208 ^g^	22.69 ± 0.081 ^f^	25.33 ± 0.021 ^e^	27.44 ± 0.070 ^d^	29.50 ± 0.031 ^C^	31.62 ± 0.035 ^b^	33.92 ± 0.055 ^a^
Zinc (mg/100 g)	1.13 ± 0.004 ^g^	1.14 ± 0.002 ^g^	1.17 ± 0.002 ^f^	1.18 ± 0.001 ^e^	1.21 ± 0.003 ^d^	1.25 ± 0.003 ^c^	1.27 ± 0.003 ^b^
Phenolic content (mg GAE/100 g)	50.25 ± 1.72 ^d^	54.63 ± 2.26 ^d^	66.68 ± 1.56 ^c^	68.25 ± 2.28 ^c^	71.50 ± 1.32 ^c^	81.75 ± 3.44 ^b^	88.75 ± 3.94 ^b^
Flavonoids (pmol QE gram)	63.65 ± 0.30 ^f^	92.06 ± 1.48 ^e^	119.60 ± 1.49 ^d^	124.54 ± 0.32 ^cd^	133.67 ± 2.50 ^c^	144.25 ± 4.13 ^b^	167.31 ± 5.23 ^a^
DPPH (% inhibition)	42.80 ± 1.76 ^c^	43.95 ± 1.82 ^bc^	44.64 ± 1.61 ^bc^	46.49 ± 0.17 ^abc^	47.76 ± 1.92 ^abc^	49.37 ± 1.30 ^ab^	50.69 ± 1.13 ^a^
FRAP (µmol TE/g)	2.52 ± 0.08 ^e^	2.84 ± 0.14 ^d^	2.96 ± 0.03 ^d^	3.78 ± 0.04 ^c^	4.56 ± 0.12 ^b^	4.62 ± 0.13 ^b^	5.12 ± 0.07 ^a^

All values are provided on dry weight basis and are average values for analysis in triplicates. Superscripts along the rows indicate significant difference (*p* < 0.05). Values in this table pertain to the composition of raw pasta.

**Table 3:** PCA results based on different nutritional parameters of different formulations of *Spirulina* biomass enriched pasta.

Attributes	Component Loading		PCA Summary Results			
	Dim 1	Dim 2	Principal Components	Eigenvalue	% Variance	% Cumulative Variance
Moisture	−0.538	0.838	Dim 1	7.921	87.651	87.651
Energy (kcal)	0.960	−0.169	Dim 2	0.91	10.070	97.720
Carbohydrates	−0.975	−0.190	Dim 3	0.159	1.759	99.480
Protein	0.996	0.064	Dim 4	0.039	0.432	99.911
Fat	0.965	0.254	Dim 5	0.007	0.077	99.989
Ash	0.920	−0.178	Dim 6	0.001	0.011	100.000
Iron	0.995	0.079				
Calcium	0.991	0.114				
Zinc	0.992	0.090				

**Table 4:** PCA results based on functional and antioxidant activity related parameters of different formulations of *Spirulina* biomass enriched pasta.

Attributes	Component Loading		PCA Summary Results			
	Dim 1	Dim 2	Principal Components	Eigenvalue	% Variance	% Cumulative Variance
Phenol content	0.974	0.119	Dim 1	3.860	96.669	96.669
Flavonoids content	0.954	0.187	Dim 2	0.103	2.579	99.248
DPPH inhibition	0.985	−0.096	Dim 3	0.027	0.676	99.924
FRAP	0.951	−0.210	Dim 4	0.003	0.075	100

**Table 5:** Cooking quality of different formulations of *Spirulina* biomass enriched pasta.

Parameter	Control	2%	5%	7.5%	10%	12.5%	15%
Cooking Time (min.)	5.35 ± 0.09 ^d^	5.35 ± 0.03 ^cd^	5.30 ± 0.06 ^bc^	5.30 ± 0.05 ^bc^	5.28 ± 0.06 ^b^	5.21 ± 0.04 ^a^	5.20 ± 0.06 ^a^
Change in Length (%)	30.97 ± 7.90 ^a^	26.13 ± 6.59 ^a^	25.92 ± 6.73 ^a^	27.03 ± 6.70 ^a^	24.98 ± 5.94 ^a^	23.76 ± 5.15 ^a^	24.67 ± 4.94 ^a^
Change in Width (%)	47.11 ± 6.88 ^ab^	59.33 ± 6.64 ^a^	40.00 ± 7.07 ^b^	58.00 ± 4.47 ^ab^	54.00 ± 8.94 ^ab^	58.00 ± 4.47 ^ab^	60.00 ± 5.00 ^a^
Gruel loss (%)	4.13 ± 0.15 ^e^	6.06 ± 0.31 ^a^	6.61 ± 0.33 ^b^	5.53 ± 0.33 ^cd^	5.13 ± 0.40 ^de^	6.66 ± 0.30 ^b^	6.22 ± 0.33 ^bc^
Increase in wt after cooking (%)	137.82 ± 3.41 ^e^	151.95 ±0.53 ^d^	161.66 ± 0.33 ^c^	162.10 ± 2.07 ^c^	182.22 ± 1.05 ^b^	190.70 ± 1.83 ^a^	193.99 ± 3.28 ^a^
Volume displacement (%)	10.00 ± 0.00 ^c^	9.67 ± 0.58 ^c^	9.33 ± 1.15 ^c^	8.67 ± 0.58 ^cd^	8.33 ± 0.58 ^cd^	8.00 ± 0.00 ^cd^	7.07 ± 0.12 ^d^

All values are provided on dry weight basis and are average values for analysis in triplicates. Superscripts along the rows indicate significant difference (*p* < 0.05).

**Table 6:** Textural attributes of different formulations of *Spirulina* biomass enriched pasta.

Level of Incorporation of *Spirulina*	Firmness (g)	Cut Force (g)	Stickiness (g.s)	Consistency (g.s)	Cohesiveness (g)
Control	4912.19 ± 201.08 ^a^	637.28 ± 27.56 ^a^	23.68 ± 1.25 ^c^	23.98 ± 2.77 ^a^	522.87 ± 16.92 ^b^
2.0%	3712.86 ± 125.56 ^b^	585.18 ± 49.81 ^ab^	41.81 ± 0.95 ^a^	14.57 ± 2.63 ^bc^	573.125 ± 18.12 ^ab^
5.0%	3689.38 ± 170.67 ^b^	562.01 ± 6.75 ^b^	39.86 ± 2.42 ^ab^	14.63 ± 2.54 ^b^	663.74 ± 11.06 ^a^
7.5%	3674.50 ± 177.73 ^b^	538.24 ± 68.91 ^b^	26.41 ± 0.57 ^bc^	11.91 ± 1.22 ^bc^	403.135 ± 12.62 ^c^
10.0%	2596.45 ± 157.36 ^c^	531.78 ± 31.10 ^c^	42.31 ± 1.90 ^a^	16.66 ± 0.23 ^b^	580.77 ± 6.41 ^ab^
12.5%	2818.56 ± 135.46 ^c^	550.02 ± 18.36 ^bc^	36.31 ± 1.62 ^ab^	13.53 ± 0.96 ^bc^	508.89 ± 19.65 ^b^
15.0%	2708.12 ± 136.76 ^c^	542.65 ± 27.46 ^bc^	25.17 ± 0.53 ^bc^	15.52 ± 1.13 ^b^	450.10 ± 14.50 ^bc^

All values are provided on dry weight basis and are average values for analysis in triplicates. Superscripts along the rows indicate significant difference (*p* < 0.05).

## Data Availability

The data presented in this study are available on request from the corresponding author.
